# Basal Cells Contribute to Pulmonary Fibrosis via SP1‐Mediated Senescence‐Associated Secretory Phenotype

**DOI:** 10.1111/crj.70202

**Published:** 2026-06-07

**Authors:** Xiaoyan Wang, Yupeng Li, Ling Zhao, Xinran Zhang, Min Liu, Huaping Dai

**Affiliations:** ^1^ Department of Respiratory and Critical Care Medicine Beijing Jishuitan Hospital, Capital Medical University Beijing China; ^2^ National Center for Respiratory Medicine, State Key Laboratory of Respiratory Health and Multimorbidity, National Clinical Research Center for Respiratory Diseases China–Japan Friendship Hospital Beijing People's Republic of China; ^3^ Department of Respiratory and Critical Care Medicine Second Affiliated Hospital of Harbin Medical University Harbin China; ^4^ Department of Pathology China–Japan Friendship Hospital Beijing China; ^5^ Department of Clinical Research and Data Management, Center of Respiratory Medicine China–Japan Friendship Hospital Beijing China; ^6^ Department of Radiology China–Japan Friendship Hospital Beijing China

**Keywords:** basal cell, idiopathic pulmonary fibrosis, senescence‐associated secretory phenotype, SP1

## Abstract

Idiopathic pulmonary fibrosis is a progressive interstitial lung disease with limited treatment options and poor prognosis. Increasing evidence suggests that airway epithelial remodeling contributes to disease pathogenesis, yet the role of basal cells remains incompletely understood. This study aimed to determine whether basal cells participate in pulmonary fibrosis through a senescence‐associated secretory phenotype regulated by the transcription factor SP1.

**Methods:**

Lung tissue from patients with idiopathic pulmonary fibrosis who underwent transbronchial cryobiopsy was compared with control samples from patients undergoing lung resection for benign nodules. Histopathological analysis and immunohistochemistry were used to evaluate basal cell distribution and quantify cell‐specific markers. Transcriptomic data from the GEO database were analyzed to identify differentially expressed genes and enriched signaling pathways. Findings were validated in a bleomycin‐induced pulmonary fibrosis mouse model, and SP1 expression was assessed by protein analysis.

**Results:**

Basal cells were markedly increased in fibrotic airways and extended from bronchioles into fibroblast foci, with their abundance correlating positively with fibrosis severity. Differential gene expression analysis identified enrichment of senescence‐associated secretory phenotype‐related pathways, including upregulation of metalloproteinases and chemokines. In the mouse model, senescence‐associated mediators were significantly elevated, and SP1 expression was increased in fibrotic lungs.

**Conclusion:**

Basal cells may actively contribute to pulmonary fibrosis, and SP1‐mediated senescence‐associated secretory phenotype may represent a potential novel pathogenic mechanism. Targeting basal cell dysfunction or SP1‐related pathways may offer new therapeutic opportunities for idiopathic pulmonary fibrosis.

## Graphical abstract

Basal cells are expanded within the small airway epithelium in IPF. This change is associated with the severity of fibrosis and may participate in disease pathogenesis via SP1‐mediated senescence‐associated secretory phenotype (SASP).

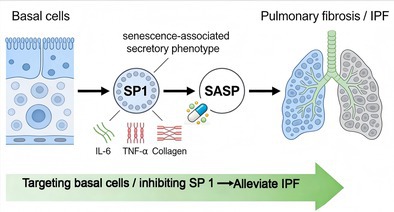

Idiopathic pulmonary fibrosis (IPF) is a progressive and fatal interstitial lung disease, marked by excessive extracellular matrix deposition, fibroblast proliferation, and destruction of alveolar architecture. It predominantly affects older men, and median survival after diagnosis is only 3–5 years despite antifibrotic therapy [1, 2, 3]. Current treatments slow but do not reverse fibrosis, underscoring the need to identify new pathogenic mechanisms.

Traditionally, IPF has been regarded as a disorder of the alveolar epithelium and interstitium. However, growing evidence suggests that the bronchial epithelium also contributes to disease pathogenesis. The MUC5B promoter variant, the strongest known genetic risk factor for IPF, drives aberrant mucus secretion in distal airways [4]. Explanted lungs demonstrate marked loss of terminal bronchioles and peribronchiolar remodeling [5], whereas structural and functional abnormalities of small airways have been observed even in early disease [6]. These findings suggest that airway epithelial cells play a critical role in fibrogenesis.

Among these epithelial populations, basal cells are of particular interest. As progenitor cells of the airway epithelium, they are essential for epithelial repair and regeneration. Basal cell hyperplasia has been noted in fibrotic lungs [7], yet their mechanistic contribution to IPF remains poorly defined. One potential pathway involves cellular senescence and the senescence‐associated secretory phenotype (SASP), through which aging epithelial cells secrete proinflammatory and profibrotic mediators. Whether basal cells in IPF adopt such a phenotype, and how this process may contribute to fibrogenesis, remains unknown.

In this study, we investigated the role of basal cells in IPF, focusing on their potential contribution through an SP1‐mediated SASP pathway. Using lung tissues from IPF patients, transcriptomic analyses of epithelial cells, and validation in a bleomycin‐induced mouse model, we examined basal cell distribution, phenotype, and regulatory mechanisms. Our findings highlight basal cells as active participants in fibrogenesis and identify SP1 as a potential upstream regulator. These results provide novel insights into epithelial–mesenchymal crosstalk in IPF and suggest potential therapeutic avenues targeting basal cell–derived SASP pathways.

## 1 Methods

This investigation was a post hoc analysis based on a prospective, multicenter cohort (NCT03666234). Patients with IPF who underwent transbronchial cryobiopsy (TBCB) at the China–Japan Friendship Hospital between June 2020 and December 2021 were **included**. The study was approved by the hospital's Ethics Committee (Approval Number: 2017‐25) and conducted in accordance with the Declaration of Helsinki. All participants (or their legal guardians) provided written informed consent. The diagnosis of IPF was established according to the American Thoracic Society/European Respiratory Society guidelines [8].

TBCB indications included interstitial lung disease with atypical high‐resolution computed tomography (HRCT) features requiring histopathologic confirmation. Procedures were performed using a 1.9–2.4 mm cryoprobe, with two to five samples obtained per patient.

**Exclusion criteria** were acute exacerbation of IPF, concomitant infection, coexisting lung cancer, or other conditions confounding the pathological interpretation.

**Control** tissues were obtained from patients who underwent segmentectomy for pulmonary nodules, with histologically normal lung parenchyma adjacent to the nodules used as controls. Groups were matched for smoking history. Preoperative pulmonary function indices were documented.

Histopathology included hematoxylin–eosin (HE) staining, Masson's trichrome staining, and **immunohistochemistry**. Fibrotic areas were quantified by Masson staining, ciliated epithelial cells by FoxJ1, and basal cells by p63. Ten random 200× fields of view per slide were scored. The immunohistochemical **score** for each field consisted of the product of the percentage of stained positive cells and the intensity score. The average of the 10 fields was calculated as the slide's immunohistochemical score. Intensity Grading: No staining: Grade 0; Pale yellow staining: Grade 1; Brownish‐yellow staining: Grade 2; Brown staining: Grade 3. Staining area and absorbance were also quantified using Image‐Pro Plus software. The pathological tissue fibrosis score was assessed according to the method described by Szapiel et al. [9].

Transcriptomic analysis was performed using the publicly available GEO dataset GSE136831 (single‐cell RNA sequencing). Differentially expressed genes (DEGs) between bronchial basal cells derived from IPF patients and healthy controls were screened using the limma package. Functional enrichment analyses were performed based on Gene Ontology (GO) and Kyoto Encyclopedia of Genes and Genomes (KEGG) databases, which highlighted signaling pathways related to the SASP and the pathogenesis of IPF.

A **bleomycin‐induced pulmonary fibrosis mouse model** was used to validate the candidate pathway. All experimental mice were male, aged 6–8 weeks, with an age difference of no more than 2 weeks. The mice were housed under 12‐h light/12‐h dark cycles, with controlled temperature (21°C–25°C), and ad libitum access to food and water. The experiments were approved by the Experimental Animal Ethics Committee of the China–Japan Friendship Hospital (Zryhyy61‐20‐10‐1). To investigate differences in cellular senescence and SASP‐related molecules between fibrotic and control lungs, further studies were performed using bleomycin‐induced pulmonary fibrosis model mice and control mice. Modeling was performed via intraperitoneal bolus injection of bleomycin. Fifty male mice aged 6–8 weeks were selected for each group. The fibrosis group received intraperitoneal injections of bleomycin (35 mg/kg dissolved in 200 μL saline) twice weekly for four consecutive weeks. The control group received intraperitoneal injections of 200 μL saline under the same conditions. Tissue samples were collected at Weeks 1, 2, 4, 6, 8, and 10 post‐modeling, with eight mice sampled at each time point. Bronchi were isolated from the lungs, and the expression levels of matrix metalloproteinase 1 (MMP1), MMP7, MMP11, C‐C motif chemokine ligand 5 (CCL5), and CCL19 were compared between the bleomycin‐induced and control groups. Using the TRRUST database (http://www.grnpedia.org/trrust/), SP1 was identified as a key regulator of multiple DEGs. SP1 expression levels were confirmed in mouse bronchial tissues by Western blot.

All **statistical analyses** were conducted using SPSS software (Version 26.0; IBM Corp., Armonk, NY, USA). Continuous variables were expressed as mean ± standard deviation, whereas categorical variables were presented as frequency and percentage. Differences in immunohistochemical absorbance between the case and control groups were assessed using Student's *t*‐test or the chi‐square test, as appropriate. Pearson's correlation and Spearman's rank correlation were applied to evaluate the association between the number of bronchial epithelial cells in IPF and the extent of local fibrosis. All statistical tests were two‐tailed, and a *p*‐value < 0.05 was considered statistically significant.

## 2 Results

### 2.1 Pathological Characteristics of Small Airways and Basal Cell Abnormalities in IPF

**FIGURE 1:**
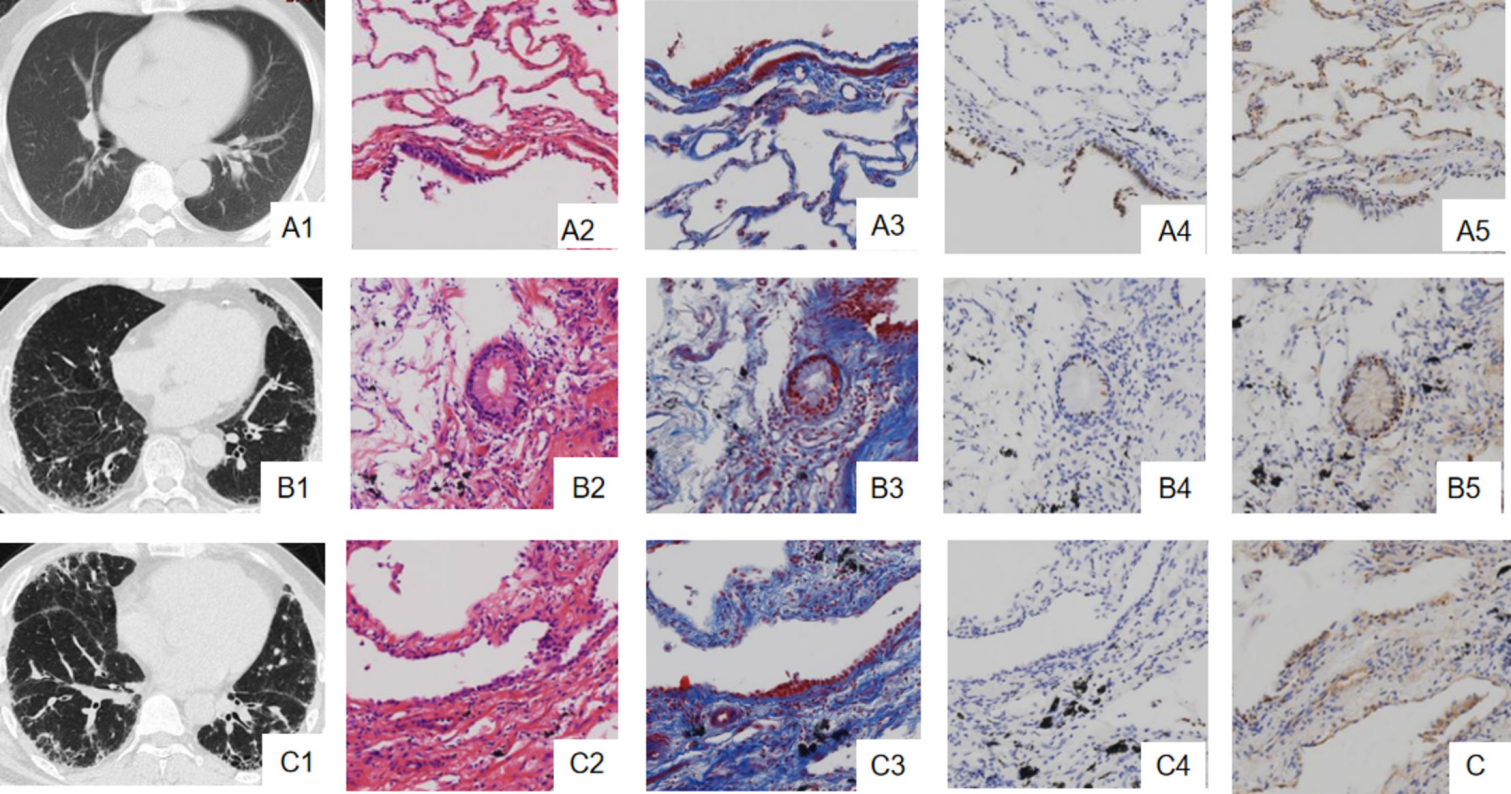
HRCT and histopathology of patients with IPF and control subjects (original magnification ×200). Each row displays sequential images from the same subject, including chest HRCT, HE staining, Masson's trichrome staining, FoxJ1 immunostaining, and P63 immunostaining.

Of 137 IPF patients, 13 underwent TBCB. As shown in Table 1, most were male (84.6%), with a mean age of 57.9 years, and 61.5% had a smoking history. Pathological examination consistently demonstrated small airway lesions, characterized by ciliated epithelial cell inversion, edema, cilia loss, and basal cell proliferation. The severity of small airway lesions paralleled surrounding alveolitis and fibrosis (Figure 1). Control subject (A1–A5): HE staining shows preserved alveolar architecture (A2). Masson's trichrome staining demonstrates absence of collagen deposition in alveolar regions, with airway epithelial cells arranged regularly without edema or hyperplasia (A3). FoxJ1 immunostaining highlights orderly, densely distributed ciliated epithelial cells (A4). P63 immunostaining shows scattered basal cells along the epithelial basement membrane without edema (A5). IPF patient 1 (B1–B5): HE staining demonstrates alveolar destruction and septal thickening (B2). Masson's trichrome staining reveals collagen deposition around small airways with epithelial edema and hyperplasia (B3). FoxJ1 immunostaining shows reduced numbers of mature ciliated epithelial cells with sparse distribution (B4). P63 immunostaining demonstrates marked basal cell hyperplasia with dense accumulation at the epithelial basement membrane (B5). IPF patient 2 (C1–C5): HE staining shows disrupted alveolar structures adjacent to small airways (C2). Masson's trichrome staining reveals collagen deposition, proliferation of airway epithelial cells, disorganized cellular arrangement, and ciliary shortening or shedding (C3). FoxJ1 immunostaining shows a further reduction in mature ciliated epithelial cells (C4). P63 immunostaining demonstrates basal cell proliferation with irregular distribution within the small airway epithelium (C5).

**TABLE 1:** Baseline characteristics comparison of IPF and control groups.

	IPF (*n* = 13)	Control (*n* = 8)	*p*
Age	57.9	59.8	0.38
Gender (male proportion)	84.6%	67.9%	0.15
Smoking history	61.5%	68.8%	0.57
HRCT pattern	UIP	Pulmonary nodules	
Sampling site	Lower lobe of the lung	The lobe where the nodule is located	

Abbreviation: UIP: usual interstitial pneumonia.

### 2.2 Absorbance Measurements Were Obtained From Immunohistochemically Stained Sections

**FIGURE 2:**
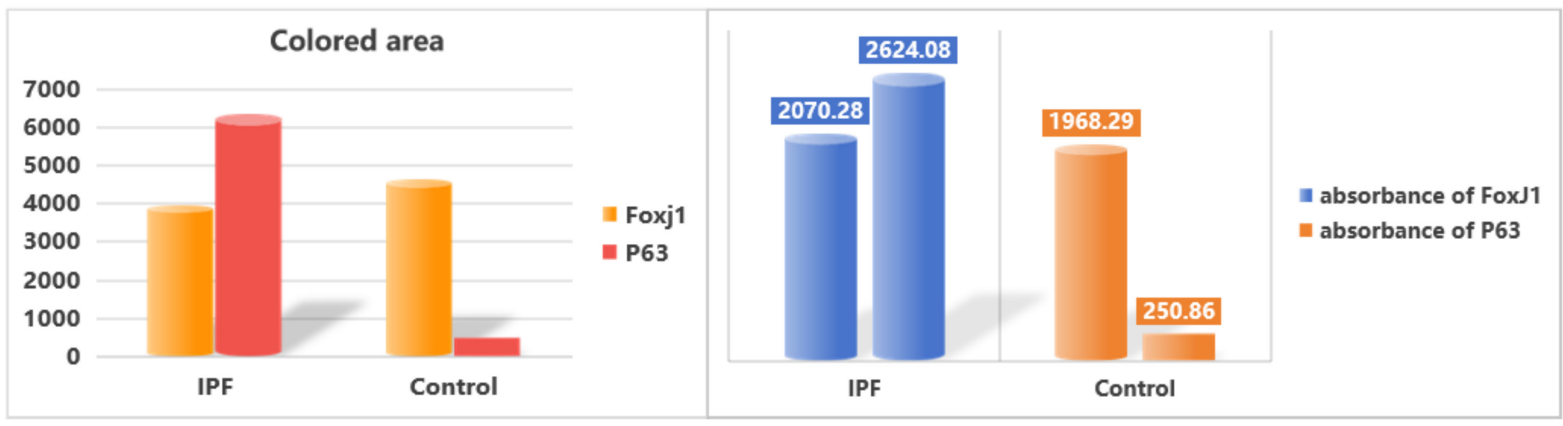
Comparison of FoxJ1 and P63 staining between IPF and control groups. (A) Staining area (μm^2^) of FoxJ1 and P63. (B) Absorbance values of FoxJ1 and P63.

In the IPF group, both the staining area (μm^2^) and absorbance of FoxJ1 showed no significant differences compared with controls (3962.24 vs. 4651.71 μm^2^; 2070.28 vs. 1968.29; *p* = 0.634 and 0.896, respectively). In contrast, the P63 staining area and absorbance were markedly increased in the IPF group compared with controls (6364.40 vs. 498.00 μm^2^; 2624.08 vs. 250.86; *p* = 0.011 and < 0.001, respectively) (Figure 2).

### 2.3 Correlation Analysis Between Immunohistochemical Staining and Fibrosis Severity in IPF

**FIGURE 3:**
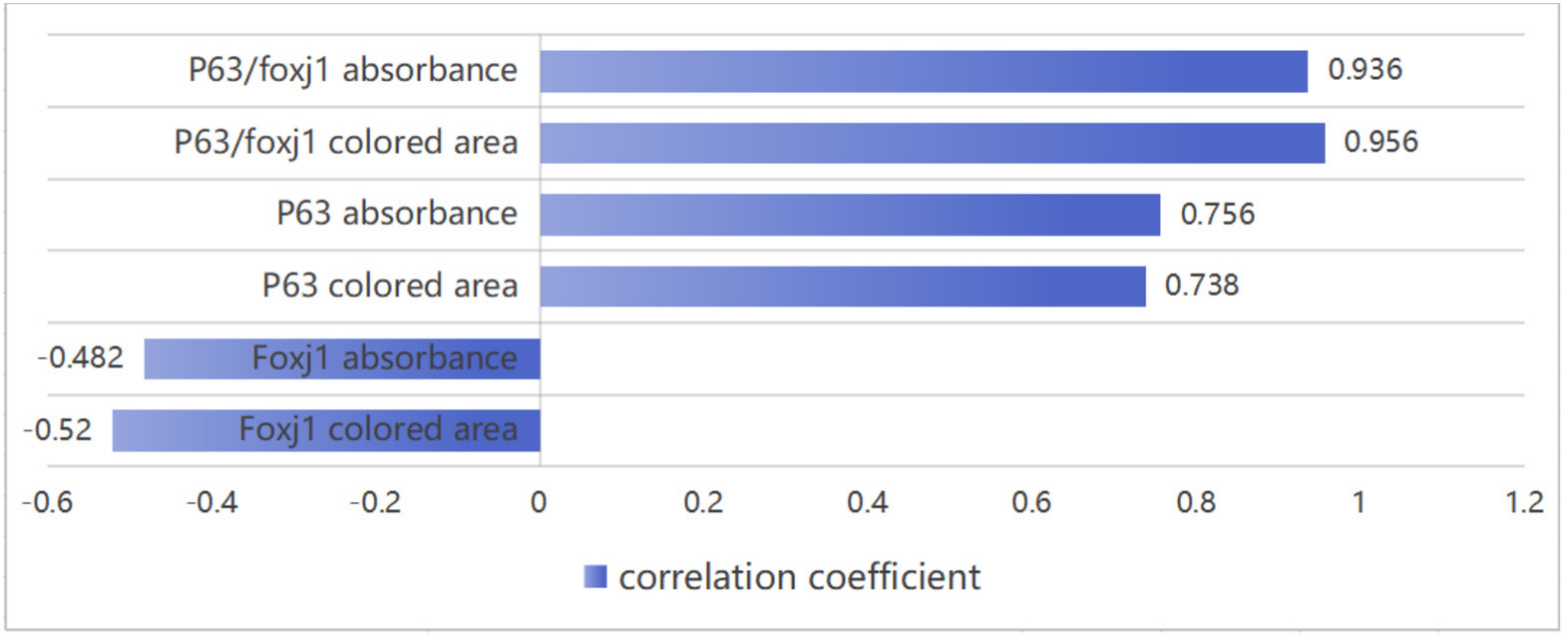
Correlation between immunohistochemical staining of bronchial epithelial cells and fibrosis severity in IPF. Shown are correlations of staining area and absorbance for ciliated and basal epithelial cells with the degree of fibrosis.

The P63 staining area and absorbance were positively correlated with the degree of fibrosis, with both *p*‐values < 0.001. The Foxj1 staining area and absorbance were negatively correlated with the degree of fibrosis, with *p*‐values of 0.002 and 0.005, respectively (Figure 3).

**FIGURE 4:**
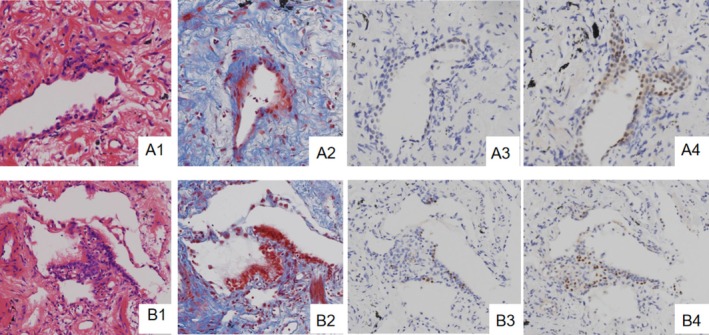
Basal cell distribution in fibrotic lesions of IPF patients. Each row displays HE staining, Masson's trichrome staining, FoxJ1 immunostaining, and P63 immunostaining, respectively. A1–A5 and B1–B5 represent histopathological sections from two IPF patients. P63 immunostaining (A4 and B4) shows brown‐stained basal cells extending from small airway walls into fibrotic areas, surrounding fibroblast foci. Original magnification ×200.

Basal cells in IPF extended from the bronchioles into fibrotic foci, suggesting their potential involvement in the fibrotic process (Figure 4).

### 2.4 SP1‐Mediated Basal Cell SASP May Contribute to the Participation of Small Airway Epithelial Cells in Fibrosis

**FIGURE 5:**
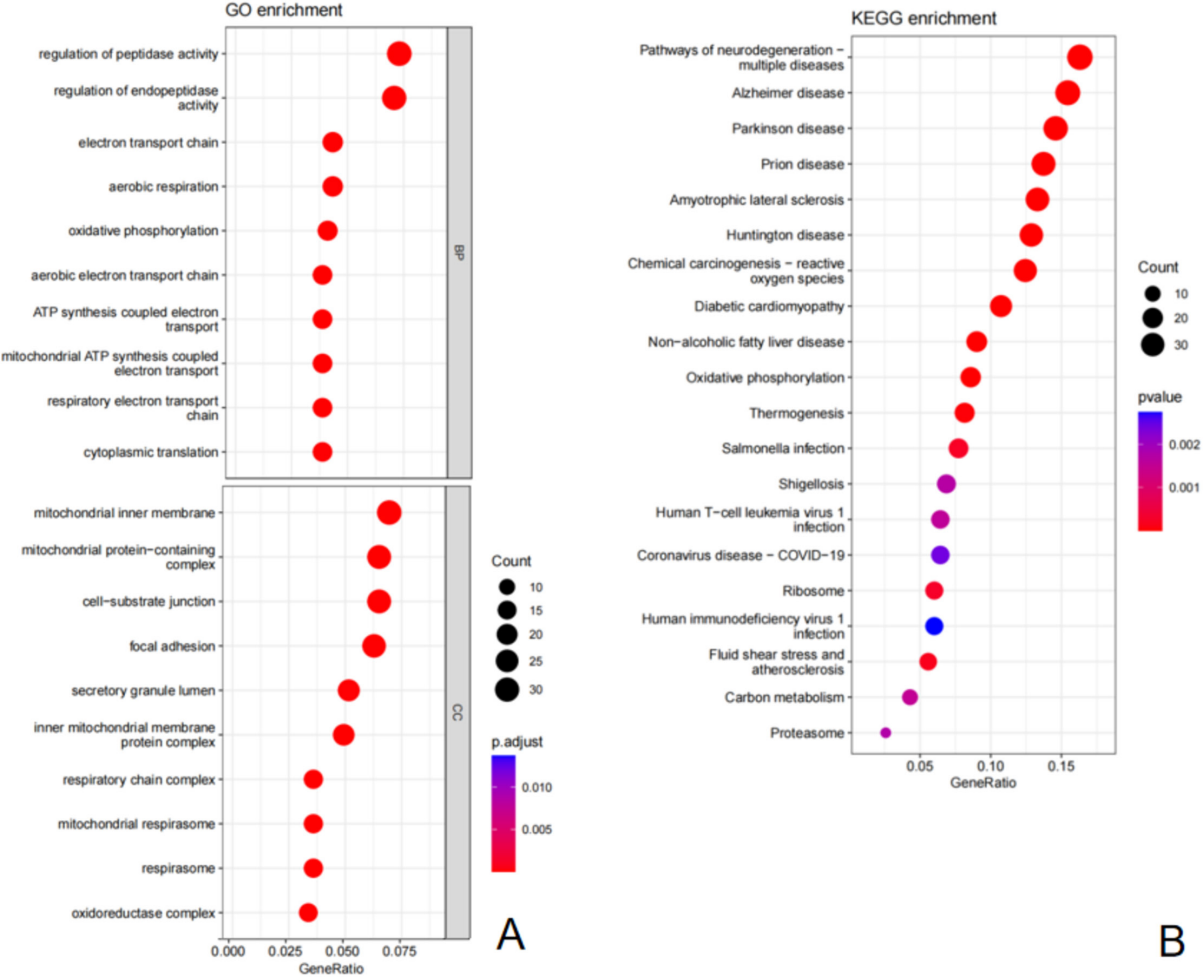
Functional enrichment analysis of differentially expressed genes in basal cells. Partial GO and KEGG enrichment results for genes upregulated in basal cells are shown, highlighting pathways associated with the SASP and IPF pathogenesis.

Single‐cell RNA sequencing data were obtained from the GSE136831 dataset in the GEO database, based on the GPL20301 Illumina HiSeq 4000 platform. DEGs between the IPF group (*n* = 14) and control group (CTR, *n* = 14) were identified using the limma package, with thresholds of log2 fold change > 1 (*n* = 170). Functional enrichment analysis of the DEGs was subsequently performed using GO and KEGG databases. Representative GO and KEGG enrichment results are shown in Figure 5.

Functional enrichment analysis of DEGs in this study revealed significant enrichment in GO and KEGG pathways related to the mitochondrial respiratory chain, oxidative phosphorylation, electron transport chain, proteasome, and ribosome. Specifically, differential expression of genes encoding components of the mitochondrial respiratory chain complexes, oxidative phosphorylation, and electron transport chain suggests substantial mitochondrial functional remodeling in basal cells. Concurrently, enrichment of pathways involved in protein translation (ribosome) and degradation (proteasome) indicates dysregulation of protein homeostasis. These enriched pathways align closely with the core mechanisms driving SASP: Mitochondrial dysfunction may trigger DNA damage response (DDR) activation via excessive reactive oxygen species (ROS) production, energy metabolism imbalance and mitochondrial DNA damage, whereas protein homeostasis imbalance (e.g., misfolded protein accumulation and endoplasmic reticulum stress) may synergistically amplify cellular stress signals, inducing cell cycle arrest and senescence, and ultimately driving transcription and secretion of SASP‐related inflammatory factors and proteases. Collectively, these findings provide critical molecular pathway‐level evidence for the activation of cellular senescence and SASP.

**FIGURE 6:**
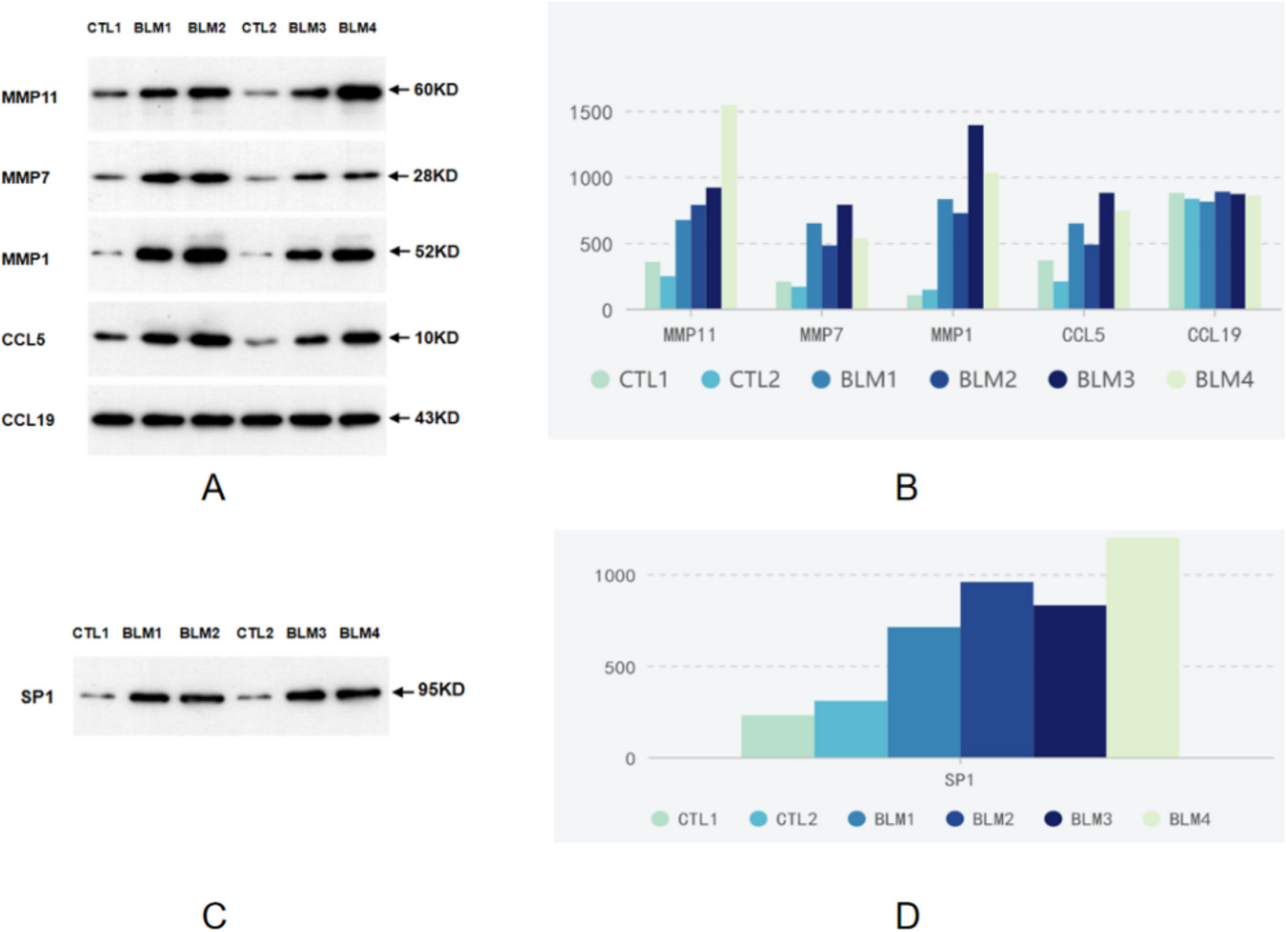
Expression of SASP‐associated molecules and SP1 in bleomycin‐induced pulmonary fibrosis mice. CTL1 and CTL2 represent control mice; BLM1 and BLM3 are bleomycin‐induced mice at the early stage of fibrosis, and BLM2 and BLM4 are mice at the fibrosis stage. (A, B) Levels of MMP1, MMP7, MMP11, CCL5, and CCL19 were elevated in bleomycin‐induced mice compared with controls. Marker expression was higher in BLM2 and BLM4 at the fibrosis stage than in BLM1 and BLM3 at the early stage. (C, D) SP1 expression in bronchi was increased in bleomycin‐induced mice relative to controls.

SASP‐associated molecules were significantly upregulated in the bleomycin‐induced group compared with controls. Bronchial SP1 expression was significantly higher in bleomycin‐induced mice than in controls, suggesting that SP1‐mediated SASP may contribute to the initiation and progression of IPF (Figures 6).

## 3 Discussion

IPF remains a devastating disease with limited therapeutic options and poor prognosis. Although historically conceptualized as a disorder of alveolar epithelial injury and fibroblast‐driven scarring, recent evidence suggests that airway epithelial biology may also play a critical role in disease progression [6]. In this study, we addressed the question of whether basal cells in the bronchial epithelium participate in fibrogenesis through an SP1‐mediated SASP. Specifically, we sought to determine whether basal cells are expanded in IPF lungs, whether their abundance correlates with local fibrosis, and whether molecular evidence supports a role for SP1‐related SASP pathways in this process. Histopathological analyses revealed marked basal cell hyperplasia in the small airway epithelium of IPF patients compared with controls, with basal cells extending into fibrotic foci and positively correlating with local fibrosis severity. Transcriptomic analysis identified DEGs enriched in SASP‐related pathways, and validation in a bleomycin‐induced fibrosis mouse model demonstrated upregulation of SASP mediators, including MMP1, MMP7, MMP11, CCL5, and CCL19. Importantly, bioinformatic analysis highlighted SP1 as a potential key transcriptional regulator of these genes, and experimental validation confirmed increased SP1 expression in fibrotic airways. Together, these data suggest that basal cells may contribute to IPF progression, at least in part through SP1‐mediated SASP activation.

These results add to a growing body of literature implicating airway epithelial remodeling in IPF pathogenesis. Gene expression profiling of bronchoalveolar lavage cells has demonstrated signatures suggestive of basal cell involvement in IPF pathobiology [10]. Several previous studies have documented basal cell hyperplasia in fibrotic lungs. Single‐cell RNA sequencing studies likewise confirm an expansion of basal cell populations in IPF lungs [11, 12, 13]. Histopathologic studies show that basal cells can extend into and accumulate within fibroblastic foci [14, 15], where they may directly secrete factors that activate fibroblasts [12]. Similarly, Adams et al. [16] observed expansion of KRT5‐positive basal cells in the distal lung regions of patients with advanced fibrosis, suggesting an aberrant repair program. More recently, basal cells have been implicated in promoting fibrosis via secretion of WNT7A, which not only activates fibroblasts but also impairs alveolar epithelial regeneration within the fibrotic niche [17]. Our study extends these observations by providing mechanistic insight: basal cells not only accumulate in fibrotic niches but also may contribute to fibrogenesis at least in part through SASP‐related signaling. Collectively, these findings underscore the multifaceted role of basal cells in epithelial–mesenchymal crosstalk and identify them as a cellular population with potential pathogenic relevance in IPF.

Beyond SASP‐related mechanisms, additional evidence further supports basal cells as potential important contributors to fibrogenesis. The role of cellular senescence and SASP in IPF has been increasingly recognized in recent years. Senescent epithelial cells secrete a variety of cytokines, chemokines, growth factors, and proteases that may contribute to chronic inflammation, fibroblast activation, and matrix remodeling [18]. Indeed, SASP factors such as MMP7, CCL2, and IL‐6 are elevated in fibrotic lungs and correlate with disease progression [19]. Our findings that basal cells in IPF exhibit a SASP‐like signature, with upregulation of multiple metalloproteinases and chemokines, provide evidence that basal cells may represent a previously underappreciated source of profibrotic SASP mediators. Moreover, the identification of SP1 as a potential central transcriptional regulator aligns with prior studies demonstrating its role in epithelial stress responses and fibrotic signaling pathways [20].

Comparing our results with prior research highlights both novelty and continuity. Whereas earlier reports have described basal cell accumulation and airway remodeling, few have directly linked basal cells to molecular pathways known to contribute to fibrosis. Our integration of histology, transcriptomics, and animal models provides a more comprehensive demonstration that basal cells are not merely bystanders but may act as active participants in the fibrotic cascade. Importantly, by implicating SP1‐related SASP, we highlight a potential therapeutic target that may be amenable to pharmacological modulation. This adds to ongoing efforts exploring senolytic therapies in fibrotic lung disease [21].

Nevertheless, several limitations of this study should be acknowledged. First, the sample size of patients undergoing TBCB was relatively small, reflecting the invasive nature of the procedure and strict inclusion criteria. Larger cohorts will be needed to further validate the generalizability of basal cell proliferation patterns. Second, the adoption of “normal” lung tissues obtained via pulmonary nodule resection as the control group has certain limitations. Pulmonary nodules are not genuine normal lung tissues, and there is a lack of direct evidence regarding whether the lung tissues adjacent to pulmonary nodules undergo structural alterations in the small airways due to the influence of nodules. Nevertheless, during tissue sampling, lung tissues far away from the nodules were selected as far as possible, generally those more than 2 cm beyond the nodule margin, to minimize the impact of pulmonary nodules on the surrounding lung tissues. Third, although transcriptomic and protein‐level analyses in both human tissues and mouse models support the activation of SASP pathways, definitive causal relationships cannot be established in this study. Functional assays, such as conditional deletion of SP1 in basal cells or lineage tracing experiments, would be required to provide more direct mechanistic evidence. Fourth, our reliance on bleomycin‐induced fibrosis as an animal model, although widely used, does not fully recapitulate the chronic and heterogeneous nature of human IPF [22].

Future studies should therefore aim to expand upon these findings through several complementary approaches. Single‐cell RNA sequencing of basal cells from IPF lungs may help yield deeper insight into their heterogeneity and dynamic states, clarifying whether specific subpopulations are responsible for SASP activation. Functional experiments, including in vitro coculture of basal cells with fibroblasts, could help elucidate the paracrine effects of SASP mediators on fibroblast activation and matrix deposition. Furthermore, genetic or pharmacological inhibition of SP1 in experimental models may clarify its potential causal role and therapeutic potential.

In **conclusion,** this study demonstrates that basal cells are expanded in the small airway epithelium of IPF lungs and correlate with fibrosis severity and may contribute to disease pathogenesis at least in part through an SP1‐mediated SASP.

## Author Contributions

X.W. designed the research and was a major contributor in writing the manuscript. Y.L. assisted with the bioinformatics analysis. L.Z. provided pathological guidance and ensured the quality of the research. X.Z. analyzed and interpreted the patient data. M.L. was responsible for HRCT reading and imaging scoring. H.D. revised the manuscript and provided the necessary research materials and equipment. All authors read and approved the final manuscript.

## Funding

This study was supported by the National Key Technologies R&D Program (No. 2021YFC2500700 and No. 2016YFC0901100 to Huaping Dai).

## Conflicts of Interest

The authors declare no conflicts of interest.

## Data Availability

The data that support the findings of this study are available upon request from the corresponding author. The data are not publicly available due to privacy or ethical restrictions.
